# Performances of the beam monitoring system and quality assurance equipment for the HIMM of carbon‐ion therapy

**DOI:** 10.1002/acm2.12916

**Published:** 2020-07-02

**Authors:** Kun Wei, Zhiguo Xu, Ruishi Mao, Zulong Zhao, Tiecheng Zhao, Qianshun She, Xincai Kang, Jianli Wang, Shengpeng Li, Min Li, Kai Song, Herun Yang, Limin Duan

**Affiliations:** ^1^ Beam diagnostic (BD) group Instituted of modern physics Chinese academic of science Lanzhou 730000 Chin

**Keywords:** beam monitoring, carbon‐ion therapy, CFDA, HIMM, quality assurance

## Abstract

**Purpose:**

The heavy‐ion medical machine (HIMM), which is the first commercial medical accelerator designed and built independently by the institute of modern physics (IMP) in Wuwei, Gansu Province, China, had officially completed clinical trials at the time of this article's writing. Three types of detector systems were developed based on the ionization‐chamber principle to monitor the beam parameters during treatment in real time, quickly verify the beam performance during a routine checkup, and ensure patient safety.

**Methods and materials:**

The above‐mentioned detector systems were used for beam monitoring and quality assurance in the treatment system. The beam‐monitoring system is composed of three integral ionization chambers (ICs) and two multistrip ionization chambers (MSICs) as a redundant design. The irradiation dose, beam position, and homogeneity of a lateral profile are monitored online by the beam‐monitoring system, and safety interlocks are established to keep the test results under the predefined tolerance limitation. The quality‐assurance equipment was composed of one MSIC and one IC stack. The IC stack was used for energy verification.

**Results:**

The off‐axis response of ICs is within a tolerance of 2%, and the dose interlock system (DIS) response time is less than 7 ms during the treatment process. The positioning resolution of MSICs reached 73 µm. The IC stack can verify the beam range within one spill and the measurement resolution is less than 0.2 mm.

**Conclusions:**

The beam‐monitoring system (BMS) and quality‐assurance equipment (QAE) have been installed and run successfully within HIMM for two years and are associated with the HIMM treatment system to deliver the right dose to the correct position precisely. Furthermore, the daily QA task is simplified by it. Above all, the system has passed the performance test of the China Food and Drug Administration (CFDA).

## 1 INTRODUCTION

Radiation therapy with carbon‐ion beams has been rapidly spreading worldwide since it was pioneered at the Lawrence Berkeley Laboratories in 1977.^1^ In contrast with conventional photon therapy, the significant advantage of carbon‐ion beams is the existence of Bragg peak, which can result in a precise conformal dose deposition to the desired volume while sparing the surrounding tissues as much as possible.^2^ On the basis of the technical and clinical research on carbon‐ion therapy at the Heavy Ion Research Facility (HIRF) in Lanzhou, China,^3^ a commercial medical accelerator with better performance than the HIRF has been designed and built independently by the Institute of Modern Physics (IMP) in Wuwei, Gansu Province, China, ie, the heavy‐ion medical machine (HIMM). The entire facility mainly consists of two electron cyclotron resonance (ECR) ion sources (the second source is a backup), a cyclotron injector, a synchrotron, and three high‐energy beam‐transport lines that deliver the accelerated carbon ions into two treatment rooms. The beam generated by the ECR ion source is preaccelerated by the cyclotron to an energy of 7 MeV/u and then injected into the synchrotron with the charge‐exchange injection scheme. The synchrotron cycle is designed to accelerate carbon ions with a kinetic energy of up to 400 MeV/u and a flux of up to 4 × 10^3^ pps.^4^ Once beams from ion sources have been accelerated to the required energy, the slow beam extraction starts, and particles are steered through the high‐energy beam‐transport line to the selected treatment room. One of the treatment rooms (Room A) is equipped with a fixed horizontal beam‐transport line, and another room (Room B) has both vertical and horizontal beam‐transport lines. In the vertical line, the beam is steered to the height of 15.5 m above the isocenter and then deflected to enter the treatment room vertically. The beam‐transport line is operated in the modulated scanning mode^5^ in treatment room A and that in treatment room B is operated in the uniform scanning mode.^6^ The relevant parameters of the HIMM facility for carbon ions are listed in Table 1.^7^, ^8^ Additionally, carbon‐ion beams have higher relative biological effects (RBEs) that can enable a superior outcome for the treatment of tumors.^2^ To give full play to the advantages of a carbon‐ion therapy device, precise dosing and irradiation position monitoring as well as robust safety interlock are all necessary conditions. Therefore, a beam‐monitoring system (BMS) and one kind of quality‐assurance equipment (QAE) were developed to confirm the beam‐delivery system sending the beam to the expected target. Research began at IMP in 1993,^9^ and during the last several years a variety of monitoring systems with specific technical characteristics have been developed to meet the needs of carbon‐ion therapy.^10^, ^11^ Currently, three types of detector systems with better performance have been developed by the beam diagnostic group of IMP for the HIMM.

**TABLE 1:** Relevant parameters of HIMM facility for carbon‐ion therapy.

Specification	Value
Treatment room	Room A, horizontal beam
	Room B, both vertical and horizontal beams
Energy range	120–400 MeV/u
Beam flux	2 × 10^6^–4 × 10^8^ pps
Radiation field	≤200 × 200 mm^2^
Dose rate	0.001–1 Gy/s
Maximum range	27 cm
Scanning technology	Room A, modulated scanning mode
	Room B, uniform scanning mode

In this paper, mainly the performance of the BMS and QAE for registration of medical devices with authorities is introduced, and using the BMS and QAE to ensure beam quality and patient safety throughout irradiation is discussed.

## 2 System Description

**FIG. 1:**
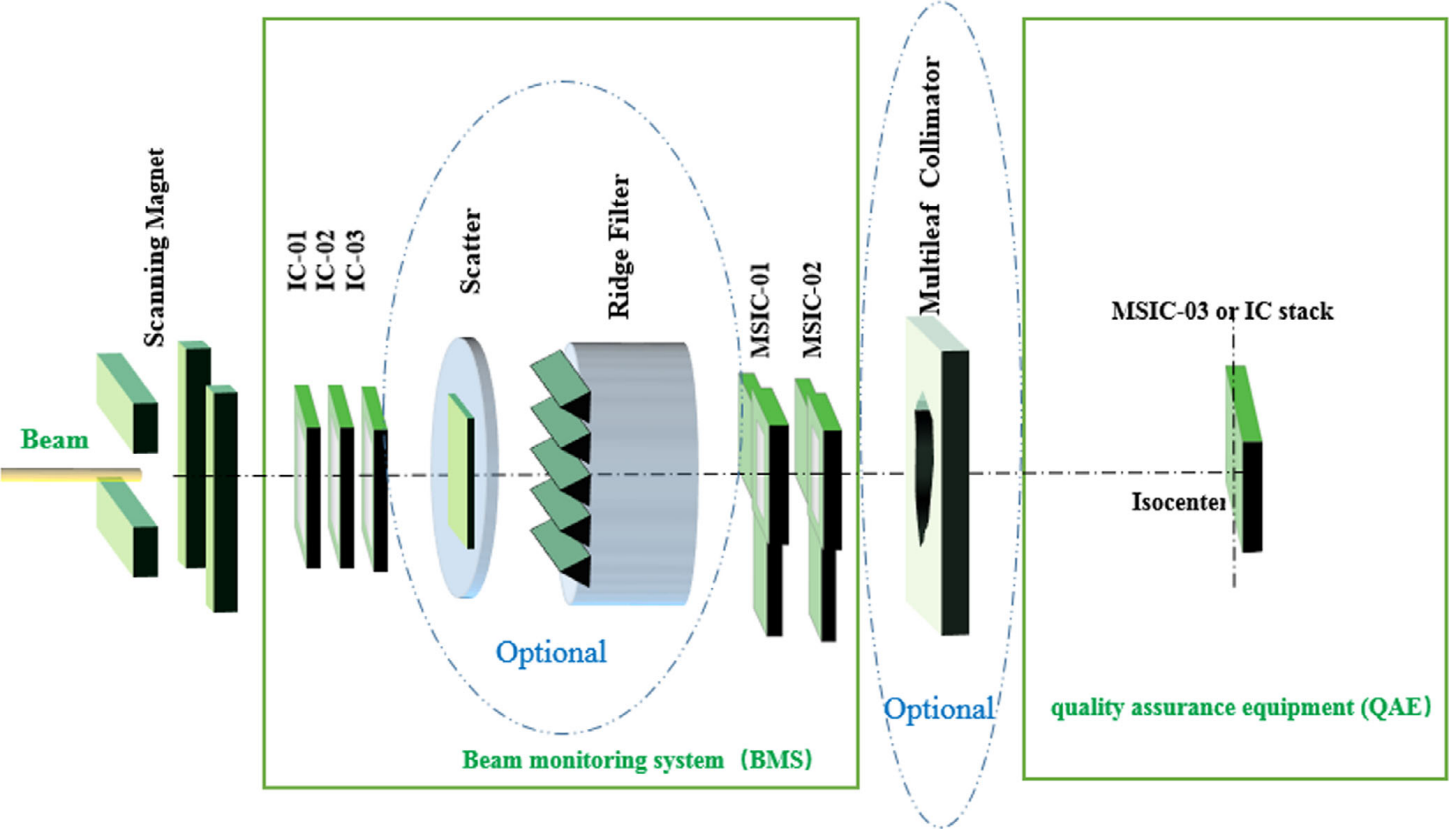
Simplified layout of BMS and QAE. The BMS is composed of three integral ionization chambers (IC‐01, IC‐02, and IC‐03) and two multistrip ionization chambers (MSIC‐01 and MSIC‐02). The QAE is composed of a multistrip ionization chamber (MSIC‐03) and an IC stack.

From the point of view of medical accelerator function, the HIMM is composed of two large systems: an acceleration system and a treatment system. The BMS and QAE are essential constituents of the treatment system that measures the beam characteristics in real time, deals with abnormal test results for patient safety throughout the treatment process, and verifies the beam parameters. According to the characteristics of the HIMM and the demands of the treatment system or physicians, three BMSs, one for each high‐energy beam‐transport line, are used for the HIMM; meanwhile, the QAE has been designed to shorten QA times. The simplified layout is shown in Fig. 1. The elements of the beam line are the BMS and a number of optional beam‐modifying elements that are chosen depending on the beam‐scanning mode.^10^, ^11^ The BMS is fixed in the beamline just behind the scanning magnets, whereas the QAE at the isocenter is portable and shared among different treatment rooms.

### 2.A Beam‐monitoring system

**FIG. 2:**
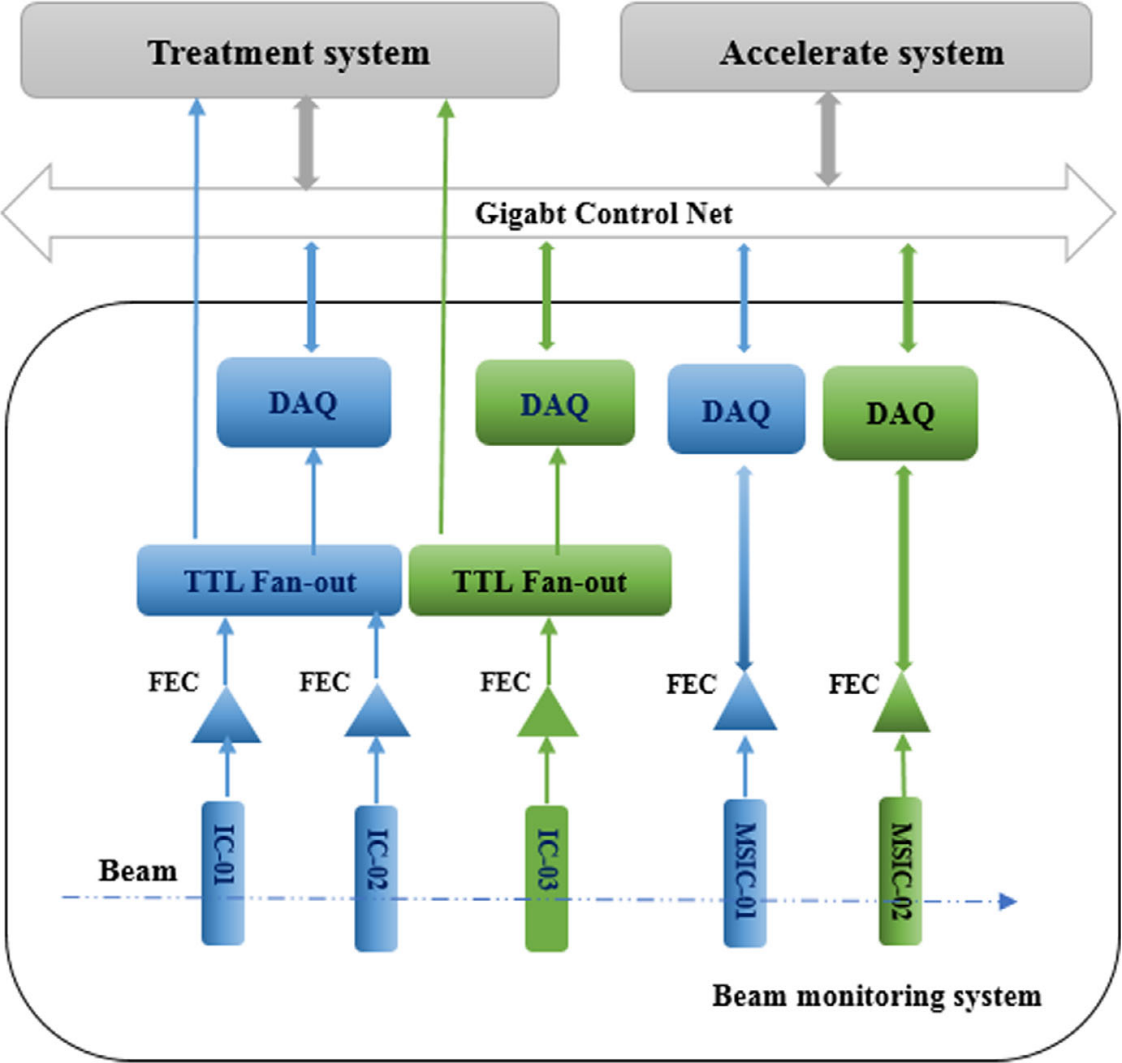
Schematic layout of BMS; the blue part represents the main system, while the green part represents the redundant system. The signal obtained from the monitors is transmitted to the accelerator system or treatment system through front‐end electronics (FEC) and a data‐acquisition system (DAQ) for beam commissioning or online monitoring during the treatment process.

The BMS is composed of three integral ionization chambers (ICs) and two multistrip ionization chambers (MSICs). For better conformal irradiation, the beam monitors located in the modulated scanning technology are used for monitoring the position of each spot and the flux delivered to each spot online; once the prescribed doses are reached, the beam is steered to the next spot by the scanning magnets. The beam current is turned on before moving the spot to the next position, until the radiation on the layer is completed, and the beam is turned off. The beam monitors placed on the uniform scanning beamline are mainly used to measure the homogeneity of the lateral beam profile and the flux delivered to the target. Meanwhile, safety interlocks are established based on the BMS to keep the test results under the predefined tolerance limitation during treatments. To reduce risk that may occur during the treatment process and meet IEC requirements for the treatment device, the BMS for each treatment line is divided into two completely independent subsystems for redundancy. The simplified schematic layout of the beam monitoring system is shown in Fig. 2, in which the blue part represents the main system and the green part the redundant system. The figure shows the communication between the BMS and the graphical user interface of the treatment system or accelerated system. The signal detected by each monitor in the BMS is sent to the treatment system; then, the treatment system analyzes the signal to select the subsequent operation.

#### 2.A.1 Integral ionization chambers

The ICs of the BMS are used to monitor the beam flux online, integrating the electrons produced by the entire lateral beam.^12^ The ICs are similar to the detector developed by IMP for clinical research of deep cancer treatment.^13^ Each IC has three electrodes made of 13‐μm‐thick Kapton® foil with an aluminum layer with a thickness of several tens of nanometers on both sides. To obtain a uniform electric field of at least 200 × 200 mm^2^, the sensitive area has been chosen to be 250 × 250 mm^2^. The collecting gap between the electrodes is 4 mm, and it is filled with nitrogen to avoid the effect of gas‐composition change. During application, the cathode is supplied with a high voltage of − 300 V; as a bias of –300 V seems small to collect all the charge, this could lead to beam‐intensity dependence. Therefore, the ICs must be calibrated at different beam intensities, and thus the calibrated coefficient was added in the data‐acquisition program for real‐time correction. The gas‐flow rate in the ICs is approximately 10 SCCM, to ensure gas tightness, the electrodes are sealed in an aluminum box, with two windows in the front and back made of 25‐μm‐thick aluminum Kapton® foil to minimize the material along the beamline.

**FIG. 3:**
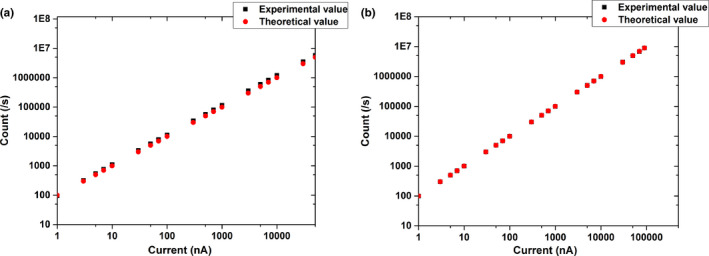
(a) Nonlinearity and (b) linearity correction of QFC output.

**FIG. 4:**
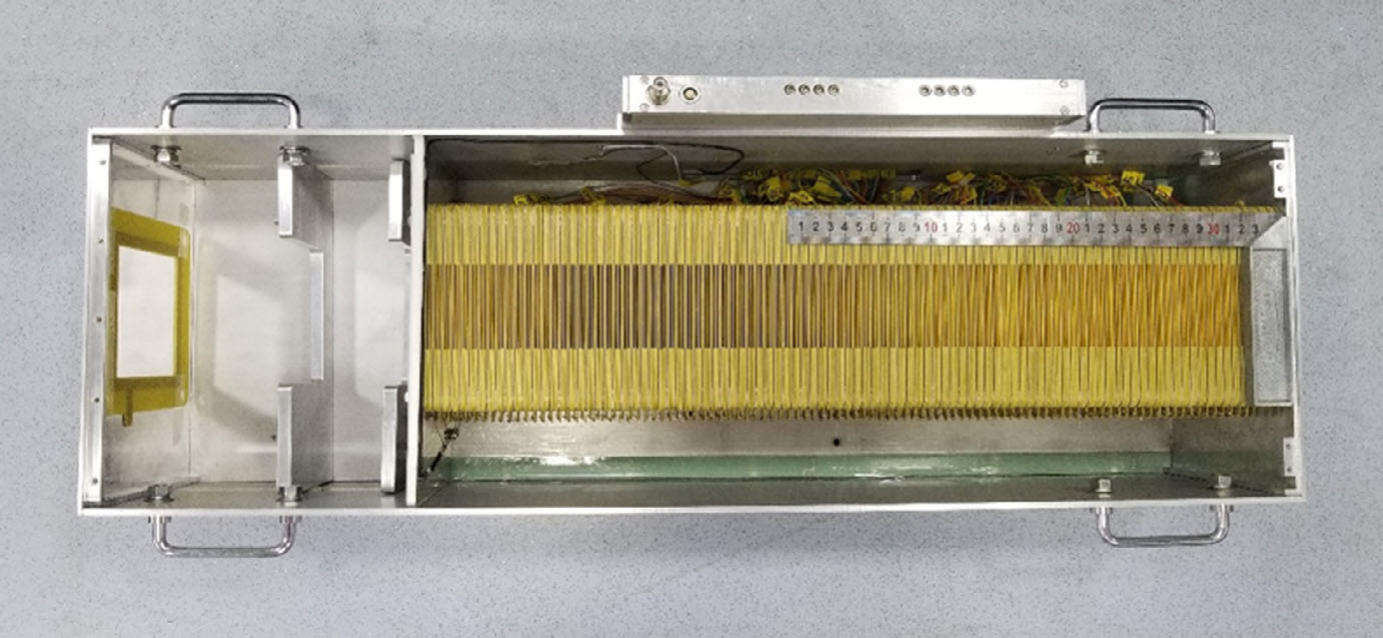
Top view of IC stack after assembly

Taking the signal range and accuracy of counts measurement into account, the front‐end electronics of the IC are based on two types of charge‐to‐frequency converters (QFCs)^14^ developed at IMP, i.e., 0.5‐ and 10‐pC/pulse QFCs. The QFCs generate digital pulses at a frequency that is proportional to the input current from the IC. The dynamic range of the 0.5‐pC/pulse QFC is between 0.05 nA and 9 μA, while that of the 10‐pC/pulse QFC is between 0.5 nA and 90 μA. Since the theoretical frequency of a QFC varies linearly with input current, a measurement on the nonlinear property of the QFC was done. A low‐noise current source, a Keithley 6221 instrument, provided the reference current to the QFC for calibration, which can determine that as the input current increases, the output frequency of the 0.5‐pC/pulse QFC increases nonlinearly in the case of small and large input‐current magnitudes, as shown in [Fig. 3(a)]. To make the experimental values approximate the theoretical values, a linear correction algorithm was implemented in the Field Programmable Gate Array (FPGA) firmware; the results are shown in [Fig. 3(b)]. In the figure, the corrected experimental values coincide well with the theoretical values. The maximum deviation is suppressed to 0.97%, which satisfies the requirement of carbon‐ion therapy Fig. 4.

The online control and data‐acquisition system of the IC is based on the National Instruments PCI Extensions for Instrumentation (PXI) platform and LabVIEW software. The signal was tested by a PXI crate with a data‐acquisition (DAQ) card NI 7841R. The maximum sampling rate of the DAQ card is 200 kHz, which can achieve data acquisition of the output signal from the QFC; the test results are transmitted to the accelerator system for beam commissioning. At the same time, the signal from the QFC is distributed to the treatment system for data processing and dose control.

#### 2.A.2 Multistrip ionization chamber

The MSIC is used to measure the beam position or homogeneity of the lateral beam profile. The information obtained from the MSIC online is one‐dimensional beam‐position information, and two‐dimensional dose distribution can be synthesized by the one‐dimensional information of each irradiation point detected by the MSIC. The prototype of the MSIC was reported elsewhere^15^; a brief description of the latest developed chamber follows. The MSIC is a parallel‐plate IC with anodes segmented in strips. The anode is made of 200 μm of FR4 (Tera‐Function epoxy with filler and fiberglass) covered with 25 μm of copper and is engraved by a standard Printed Circuit Board (PCB) technique to obtain 200 strips (0.8 mm wide with a pitch of 1 mm) and has an area of 200 × 200 mm^2^. The detector has three cathodes and two strip anodes that are oriented along the vertical and horizontal directions. The cathodes are made of 13‐μm‐thick Kapton® foil with aluminum layers of several tens of nanometers on both sides. The gas gap between each anode and cathode is 4 mm wide and polarized at − 300 V during application. The MSICs must also be calibrated at different beam intensities, and thus the calibrated coefficient was added into the data‐acquisition program for real‐time correction.

The front‐end readout circuit of the MSIC is based on the gated current integrated circuit developed by IMP,^15^ which converts the vertical (strip X) and horizontal (strip Y) charge signal outputs from the MSIC to a differential voltage signal. The promoted properties of the front‐end electronics are given in Table 2. The MSIC is connected to four 100‐channel front‐end electronics through flexible connection boards. To avoid radiation damage, the front‐end electronics are protected by several polyethylene blocks.

**TABLE 2:** Main properties of front‐end electronics.

Specification	Value
Channels	100 positive input channels
Integral capacitance	10 pF
Dynamic charge range	0 − 50 pC
Noise	<2 mV
Integral time range	minimum of 50 μs
Maximum linear error	Less than 1%

For the MSIC, a FPGA board with a DAQ card was developed for online control and data acquisition. Two software components were developed in LabVIEW for the MSIC to display in real time the position and size of the pencil beam or the beam width and homogeneity of the lateral diffusion beam in the transverse plane through strips X and Y respectively. The MSIC can complete one beam‐profile test in 100 µs. The monitored information was compared with the planned range from the treatment system, and the interlock system would be triggered when the monitored information was outside the planned range.

### 2.B Quality assurance equipment

For clinical carbon‐ion therapy facilities, a range of essential daily QA checks is carried out to verify a number of aspects of the clinical beam. This takes a great deal of time. For this purpose, a self‐developed QAE was developed for the HIMM. The QAE is composed of a MSIC and an IC stack. The MSIC with the same structure as that used in the BMS is used not only for beam‐position and beam‐width checks but also for testing beam‐angle distribution and for verifying two‐dimensional dose distribution at the isocenter. The IC stack was assembled for the purpose of energy verification a few minutes before treatment at the isocenter.

#### 2.B.1 IC stack

The IC stack is composed of 98 plane ionization chambers (PICs) and corresponding absorbing plates. The structure of the PIC is the same as that of the IC mentioned in Section 2.1.1, and the sensitive area of these chambers measures 100 × 100 mm^2^. Since polymethyl methacrylate (PMMA)^16^ (density 1.19 g/cm^3^, electron density 3.7 × 10^29^ m^−3^) is an excellent water‐equivalent (density 1.00 g/cm^3^, electron density 3.0 × 10^29^ m^−3^) material, a 1‐mm‐thick layer of PMMA was fixed in front of every PIC as an absorbing plate to simulate different water depths. During application, the PICs were filled with air at atmospheric pressure for more comfortable operation, and the cathodes were supplied with a 300‐V negative voltage. By measuring the signal from the anode of the 98 PICs synchronously, a discretized approximation of the theoretical Bragg curve could be obtained, as well as the maximum signal position.

Since a discretized Bragg curve must be obtained, the electronics must be able to individually collect the integrated charges measured by all the PICs and integrate each of the chamber's signals synchronously. For this purpose, the front‐end electronics of the IC stack detector are based on a gated current integrator circuit as well; it samples the output signals from the 98 PICs synchronously through a cable. The signals from the PICs (several nC/s to tens of uC/s) are converted into voltage signals (0 − 1 V) by the front‐end electronics.

The data‐acquisition system is based on NI CompactRIO (CRIO). The data obtained from the NI 9220 and NI 9402 acquisition cards are analyzed by LabVIEW software. Since the maximum sampling rate of the NI 9220 is 100 Ks/s, the beam energy can be verified within 1 ms. During application, the background current of electronics can be modified in the acquisition program by subtracting the baseline value when no signal is inputted.

## 3 Performance Tests

The performance tests of the BMS and QAE were conducted under different irradiation conditions during the preclinical beam‐commissioning phase. The test results were found to satisfy the requirements of carbon‐ion radiotherapy. Details of the main tests are presented in this section.

### 3.A Off‐axis response of ICs

**FIG. 5:**
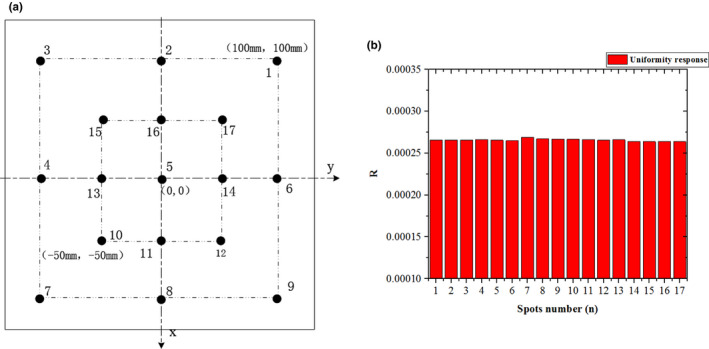
(a) Spot distribution for off‐axis test. (b) Relative dose response in treatment room A.

The ICs must ensure that the beam intensity measured by the effective volume responds uniformly. To avoid overdose or underdose irradiation, the spacing uniformity between the electrode plates of the ICs is required to be better than 0.2 mm/cm and the off‐axis response of ICs should be limited to ± 2%. To test the off‐axis response of the ICs, 17 spots with the same beam parameters arranged as shown in [Fig. 5(a)] were delivered at the isocenter. The dose collected by an external standard ionization chamber (PTW‐Freiburg) and put in the air, moved spot by spot synchronously with the beam, was used as a reference to measure the relative dose in each point. As defined by the IEC, the off‐axis response of ICs is expressed as

(1) $$D=\left(\frac{\left|R\text{max}\;-\;\text{Rmin}\right|}{\text{R1}}\right)\times 100\%,$$

where $R_{\max}$ and $R_{\min}$ are the respective maximum and minimum ratios of the dose measured by the PTW‐Freiburg unit to the counts measured by the ICs synchronously; $\bar{R}$ is the average value of the ratios *R*. The off‐axis value for 17 spots was found to be within the ± 2% tolerance for all ICs. For example, the relative dose response (R) tested by IC01 with a beam energy of 261.3 MeV/u in treatment room A is shown in [Fig. 5(b)], and the maximum differences between the maximum and minimum values of the ratio $\bar{R}$ are 1.98%. Since random errors are introduced in the testing process, the off‐axis value should be better than this value.

### 3.B Sensitivity of MSIC

**FIG. 6:**
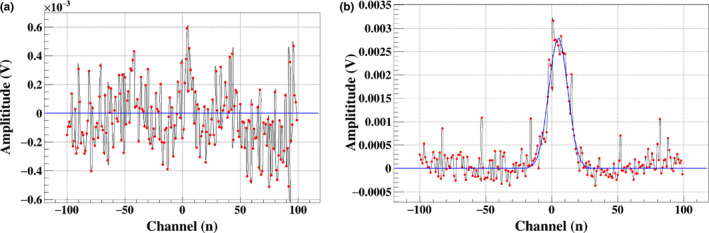
(a) Noise of MSIC with baseline subtraction. (b) Minimum distinguishable signal.

The sensitivity of the MSICs is defined as the minimum signal that can be detected; signals below this sensitivity cannot be detected, thereby causing overdose irradiation. The sensitivity of the MSIC is affected by the background current of detectors, front‐end electronics, and data‐acquisition systems. To obtain accurate data results, special care was devoted to limiting the background current to acquire enough signal‐to‐noise ratio (SNR) as it may affect the dose measurements, especially for low beam intensities. Before irradiation, the background current of the electronics can be modified in the acquisition program by subtracting the baseline value when no signal is inputted; for example, the modified counts per channel of the MSIC are shown in [Fig. 6(a)], in which the baseline drift caused by temperature change is nearly eliminated. Figure 6(b) shows that the minimum distinguishable amplitude is approximately 2.5 mV in 50 µs and the dose error caused by the signal below 2.5 mV is less than 0.06 cGy. Therefore, the beam parameters could be monitored by the MSIC at low dose rate, which reduced the effect on the patients.

### 3.C Position resolution of MSICs

**FIG. 7:**
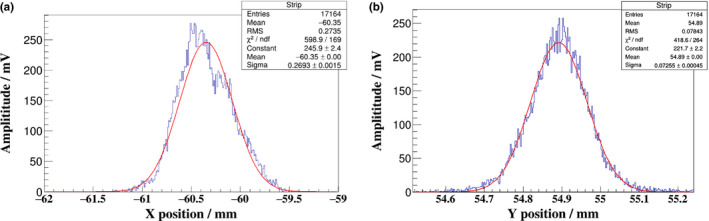
Position resolution of MSIC in X and the Y directions.

The position resolution of the MSICs is limited to the accuracy of the strip anode and the performance of the front‐end electronics. It was tested by irradiating spots with a sigma value of approximately 3 mm and 5 × 10^4^ carbon ions. According to the analysis of the data obtained from the same point repeatedly, it is found that the sigma values of the beam‐spot‐position distribution of the X and Y directions were 269.3 and 73 μm respectively. From the test results of position resolution in two directions, the beam oscillating at a fixed frequency in the X direction was found; this problem was solved during the accelerator‐commissioning process. Therefore, the position resolution of the MSIC reaches 73 μm without consideration of the beam stability Fig. 7.

Since treatment accuracy refers to the difference between beam‐radiation location and plan location, it is worth mentioning that an MSIC used at the isocenter can improve treatment accuracy before treatment because the position resolution of the MSIC is the main influencing factor of treatment accuracy measurement.

### 3.D Homogeneity of lateral beam profile

**FIG. 8:**
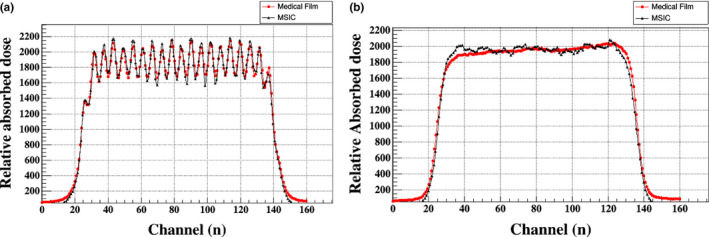
Field profiles along the (a) X and (b) Y axis measured by MSIC and a medical film.

The homogeneity of the lateral beam profile is measured for QA by the MSIC or medical film for irradiating a specific treatment field. To confirm that the MSIC is good at measuring the homogeneity, an EBT3 medical film was attached to the front window of the MSIC to test the beam profile synchronously. The measured field profiles along the X and Y axes for a square field of 110 × 110 mm^2^ irradiated with 2 × 10^7^carbon ions (330 MeV/u) and a ridge filter^17^ are shown in Fig. 8. In our beam‐delivery system, the ridge filter is employed as the range modulator.

It can be seen from the test results that the picket of the ridge filter measured by the MSIC is well consistent with the EBT3 (resolution 72 dpi) medical film; the difference is mainly found in the picket of the ridge filter, i.e., the maximum deviation is 8.8% after data normalization. Through consulting literature and data analysis, it was determined that the difference is mainly caused by the nonlinear relationship between the beam flux and the medical film absorbed dose.^18^, ^19^ The test results prove that the commonly used medical film can be replaced by the MSIC in measuring the homogeneity of the lateral beam profile. Furthermore, since the EBT3 image must be scanned and analyzed offline, the MSIC can achieve faster measurement than medical film; this feature is more conducive to clinical daily QA and for identifying and solving problems in a timely manner.

### 3.E Interlock response time

The safety of treatment mainly relies on two interlock systems to detect abnormal conditions and interrupt beam delivery: a dose interlock system (DIS) and position interlock system (PIS). Accounting for the latency of detectors, front‐end electronics, and the beam‐extraction system, the interlock is not able to shut down the beamline immediately when the threshold is reached. Therefore, the overdose irradiation caused by interlock response time should be considered in the treatment process.

**FIG. 9:**
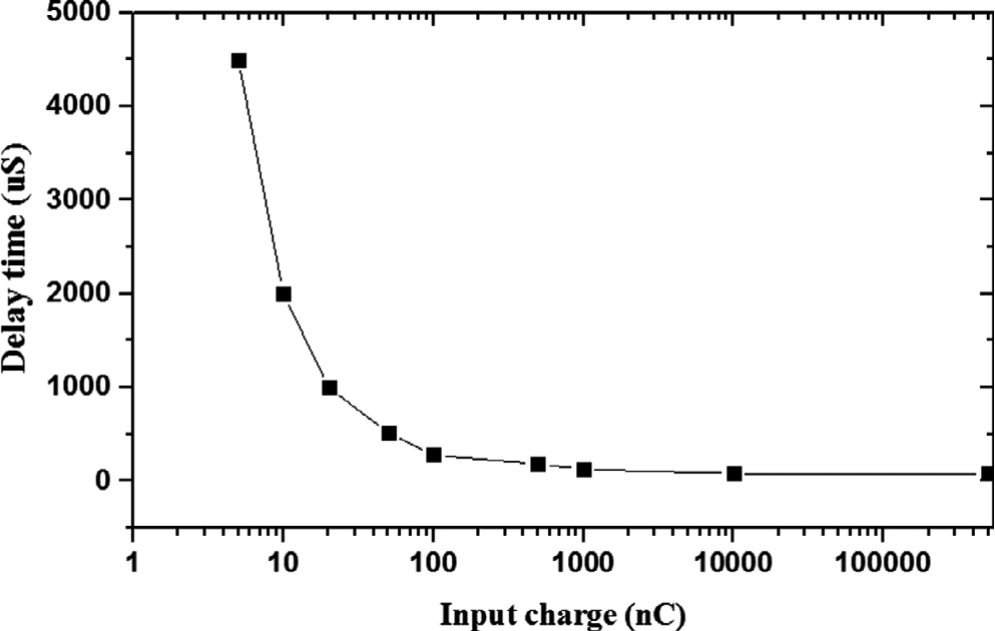
Relationship between response time of QFC (0.5 pC/pulse) and input signal from IC.

The working principle of the DIS is based on the counts measured by the IC. The monitored counts are compared with the test plan in the treatment system, and then the treatment system sends the interlock signal to the slow beam‐extraction system to interrupt the beam through an optical signal as soon as the measured counts exceed the predefined tolerance bands. The response time of the IC can be simulated by Garfield++,^20^ and the result is approximately 10 μs during application. The response time of the QFC (0.5 pC/pulse) is shown in Fig. 9, and it decreases with increasing input signal from the IC. Since the charged signal of the IC reaches up to 10^4^ nC, the response time of the QFC is approximately 100 μs during application. To measure the entire response time of the DIS, experiments were conducted by setting the interlock dose to 0 cGy and recording the response time and counts measured by the IC. Test results show that the response time of the DIS is less than 7 ms with a beam energy of 120 MeV/u and beam flux of 4 × 10^8^ pps; the resulting counts tested by IC are less than 144, which is approximately 0.15 cGy when converted into dose. This result may help control overdose irradiation in the treatment process.

The PIS will be triggered in the case of the position or homogeneity of the lateral beam profile tested by the MSIC being outside the predefined tolerance bands, or the position difference or homogeneity difference of the lateral beam profile tested by MSIC‐01 and MSIC‐02 exceeding the threshold. The abnormal or error conditions detected by the MSIC were sent to the treatment system; then, the treatment system sent the signal to the slow beam‐extraction system to interrupt the beam through an optical signal to guarantee a quick reaction. The response time of the PIS is approximately 2–3 ms shorter than that of the DIS under the same condition. Since the maximum dose rate of the HIMM is 1 Gy/s, the calculated overdose irradiation is approximately 0.09 cGy.

### 3.F Beam‐angle measurement

The beam angle can be obtained by measuring the relative beam positions along the beamline from the MSICs in a different location. Thus, we define the beam angle as

(2) $$\tan\theta =\frac{R}{L},$$

where *R* is the longitudinal difference between the beam position measured by the MSICs in a different location and *L* the distance between the two MSICs.

**FIG. 10:**
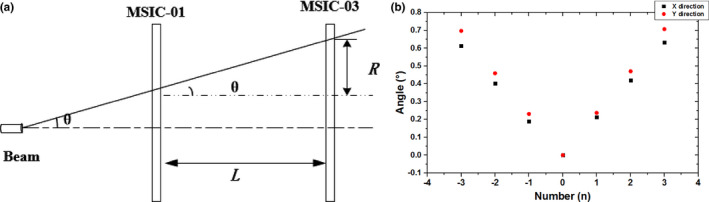
(a) Schematic of test. (b) Beam‐angle distribution with change of spot number (beam position) in X and Y directions.

The beam angle was tested by irradiating a 7 × 7 grid of spots with the same spacing from each other, and the beam position was measured by MSIC‐01 and MSIC‐03 (placed at the isocenter) synchronously. Since the distance between the two MSICs is 1,750 mm, the distribution of the beam angle can be obtained by substituting corresponding parameters in formula (1). The beam‐angle distribution with the change of spot number (beam position) in the X Y directions is shown in Fig. 10; as the beam position deviates from the central location, the beam angle increases linearly. From this result, the beam position at the isocenter can be calculated through the beam position measured by MSIC‐01, which can simplify the QA process.

### 3.G Beam‐range verification

**FIG. 11:**
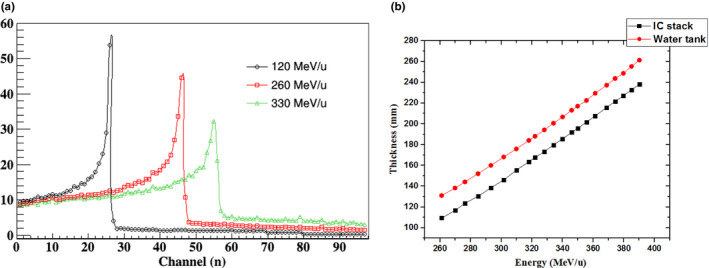
(a) Depth dose distribution measured at different energies. (b) Relationship between beam energy and peak position of Bragg curves tested using an IC stack and water tank.

Since a 1‐mm‐thick layer of PMMA was fixed in front of every PIC as an absorbing plate to simulate different water depths, the total of 11.66 cm of equivalent water thickness determines that the IC stack cannot measure the entire depth of the dose distribution. Therefore, different thicknesses of PMMA were added in front of the IC stack to make the peak position of the Bragg curves appear in the sensitive area of the chamber. As an example, for irradiation with 120‐, 260‐, and 330‐MeV carbon‐ion beams, 1, 70, and 120 mm, respectively, of PMMA was added in front of the IC stack. The depth dose distributions measured by the IC stack are expressed by dots in [Fig. 11(a)]. Since the beam‐energy loss varies slightly in the flat area, the loss of partial flat‐area information has an ignorable effect on the measurement of depth dose distribution in the Bragg‐peak region. The relationship between beam energy and the peak position of the Bragg curves tested by the IC stack can be seen in Fig. 11(b); compared with the measured results from a water tank, it is found that the linearity is basically the same.

**FIG. 12:**
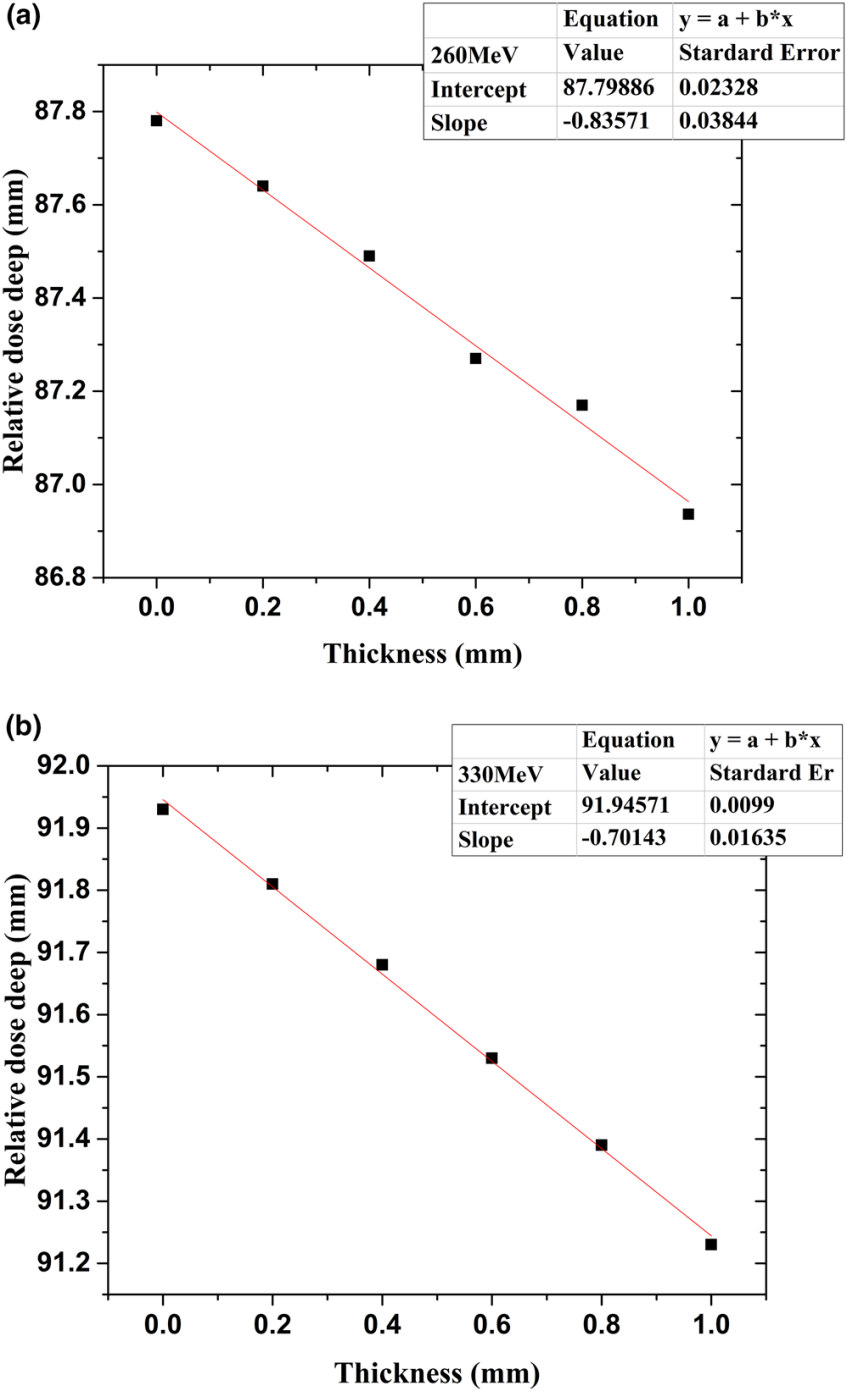
Relationship between relative position of Bragg peak and increased PMMA thickness: (a) 260 and (b) 330 MeV.

The accuracy of depth dose distribution in the Bragg‐peak region was tested by adding PMMA with a thickness of 0.2 mm layer by layer in front of the IC stack. With the increase in the number of 0.2‐mm‐thick layers of PMMA, the relative position of the Bragg peak changed; the changes in position can be calculated accurately by the weighted‐mean‐center method^21^. Figure 12 shows the relationship between the relative position of the Bragg peak and the increased PMMA thickness. It was determined that as the PMMA thickness increases the relative position decreases linearly. Since the approximate algorithm used to calculate the Bragg‐peak position vs PMMA thickness is related to the depth dose distribution, the slopes of different energies are different, but the accuracy of the specific energy that is calibrated using a water tank is not affected. The linear deviation is less than 0.04 mm with a beam energy of 260 MeV and less than 0.02 mm with a beam energy of 330 MeV, which shows the superior performance of the IC stack.

## 4 DISCUSSION AND CONCLUSIONS

The BMS and QAE that were specially developed for the HIMM facility were installed and run successfully within the HIMM for two years. Taking full advantage of the BMS and QAE, the accuracy of dose delivery is better than IEC requirements, and the treatment efficiency has clearly increased. In particular, the beam oscillating within a range of approximately 0.35 mm caused by a scanning‐magnet power source was found from the test results of MSIC; this was solved during the accelerator‐commissioning process. The performance of the BMS and QAE was tested with different irradiation conditions during the preclinical beam‐commissioning phase, and some examples are reported in this paper. The test results proved that the BMS and QAE satisfy the requirements of the carbon‐ion radiotherapy with a high level of reliability. The BMS and QAE associate the treatment system of the HIMM to deliver the right dose to the correct position precisely. Furthermore, it simplifies the daily verification task.

## CONFLICT OF INTEREST

The authors have no conflict of interest to disclose.
